# Outcomes of cardiac arrest hospitalizations in patients with obesity with versus without prior bariatric surgery status:A nationwide propensity-matched analysis^☆^

**DOI:** 10.1016/j.ijcrp.2023.200235

**Published:** 2023-12-22

**Authors:** Rupak Desai, Zainab Gandhi, Abhimanyu Ravalani, Kamran Mahfooz, Uvesh Mansuri, Akhil Jain, Ankit Vyas, Rajeev Gupta, Carl J. Lavie

**Affiliations:** a Independent Researcher, Atlanta, GA, USA; b Department of Internal Medicine, Geisinger Wyoming Valley Medical Center, Wilkes Barre, PA, USA; c Department of Internal Medicine, Saint Agnes Hospital, Baltimore, MD, USA; d Department of Internal Medicine, Lincoln Medical Center, New York, NY, USA; e Department of Internal Medicine, Medstar Harbor hospital, Baltimore, MD, USA; f Department of Internal Medicine, Mercy Catholic Medical Center, Darby, PA, USA; g Division of Vascular Medicine, Ochsner Health, New Orleans, LA, USA; h Consultant Cardiologist, Spectrum Medical Center and Burjeel Royal Hospital, Al Ain, United Arab Emirates; i John Ochsner Heart and Vascular Institute, Ochsner Clinical School, The University of Queensland School of Medicine, New Orleans, LA, USA

**Keywords:** Cardiac arrest, Mortality, Obesity/obese, Bariatric surgery, Weight loss surgery

## Abstract

**Introduction:**

Prior bariatric surgery (PBS) status in obese patients is thought to curtail the risk of cardiovascular events, but its role in change of outcomes of patients with obesity developing new acute cardiac events such as cardiac arrests (CA) remains largely unknown.

**Methods:**

Hospitalizations among adult patients with obesity and CA were identified retrospectively using the National Inpatient Sample (2015 October-2017 December). Propensity-matched analysis (1:1) was performed for sociodemographic/hospital characteristics to identify two cohorts, with (PBS+) or without (PBS-) status. The primary endpoint was in-hospital mortality, and the secondary endpoint was healthcare resource utilization.

**Results:**

Both cohorts (n = 1275 each), had patients with comparable age (mean 58 years), with a higher frequency of white (>70 %), females (>60 %), and Medicare enrollees (>40 %). PBS + cohort had lower rates of diabetes (27.8 % vs 36.1 %), hyperlipidemia (33.7 % vs 48.6 %), renal failure (17.3 % vs 22.0 %), chronic pulmonary disease (11.8 % vs 21.2 %) and higher rates of anemias (18.4 % vs 12.2 %), liver disease (5.1 % vs 2.4 %) and alcohol abuse (6.7 % vs 2.4 %) than PBS- cohort (p < 0.05). All-cause mortality (46.3 % vs 45.1 %, p = 0.551) was comparable between the two cohorts. The PBS + cohort was less often transferred routinely (p<0.001) but had a shorter hospital stay (p<0.001) with equivalent hospital charges compared to the PBS- cohort.

**Conclusions:**

The PBS status (regardless of chronology) did not increase survival in CA admissions among patients with obesity. Preventive measures are necessary to manage enduring cardiovascular disease risk factors that may limit the advantages of surgery for patients with obesity and aggravate the worse outcomes of future cardiac events.

## 1 Introduction

Metabolic and Bariatric surgery (BAS) has been claimed to have benefits beyond weight loss on metabolic syndrome, type 2 diabetes mellitus and cardiovascular disease (CVD) and mortality [1]. Retrospective studies have shown improvement with a risk reduction of 82 % in CVD burden with prior BAS (PBS) and mortality benefit for up to 56 % from CVD after BAS [2,3]. Sjostrom et al. studied long-term CVD outcomes in patients with obesity after BAS and found a decreased incidence of deaths from acute myocardial infarction when followed for up to 20 years by a hazard ratio of 0.67 [4]. We focus on cardiac arrest (CA) as an outcome of interest due to the staggeringly low survival rate of mere 10 % with OHCA (out of hospital CA) and 49 % with IHCA (in-hospital CA) across the United States. While initial survival may be higher but survival rate at 3 months after IHCA ranges between 13 and 27 % and at one year is about 13 % [[5], [6], [7]]. Intuitively, IHCA tends to have better outcomes given the resource availability and expertise; however, patient factors affecting outcomes need further analysis and remain largely unexplored. Thus, we aimed to analyze whether while the incidence and burden of CVD tend to improve with BAS in the obese population, BAS status can affect mortality outcomes of CA hospitalizations in patients with obesity using a nationally representative sample in the United States.

## 2 Methods

### 2.1 Study population

The National Inpatient Sample (NIS) which is the largest publicly accessible all-payer inpatient database in the US as a part of the Healthcare Cost and Utilization Project (HCUP) was queried from October 2015 to December 2017 [8]. Since NIS is a de-identified database, institutional review board approval was not required. In this retrospective observational analysis, adult CA hospitalizations were identified with the International Classification of Diseases Tenth Revision, Clinical Modification (ICD 10-CM) diagnostic code I46. x and obesity cases were coded using Elixhauser Comorbidity Software. The ICD-10-CM code Z98.84 was used to identify the PBS status.

### 2.2 Study group and outcomes of interest

Two cohorts were divided into the sample population: hospitalizations with PBS (PBS +) and without PBS (PBS -). Patient and hospital-level characteristics and pre-existing comorbidities were compared between cohorts using relevant ICD –10 codes for each comorbidity.

The primary outcomes were all-cause mortality and secondary outcomes were utilization of health care resources including patient disposition (routine, short-term hospital transfer, skilled or intermediate nursing facility, and other transfers), length of stay (LOS), and cost of care (as per 2017).

### 2.3 Statistical analysis

Weighted discharges and complex survey modules with IBM SPSS Statistics version 25.0 (IBM Corp., Armonk, NY, USA) were used for all analyses. We used Pearson chi-square test for categorical measures and Mann Whitney *U* test for continuous variables (non-normal distribution). A two-tailed *p* < 0.05 was considered a threshold for clinical significance. Due to a substantial difference in the total number of valid observations between the two groups of all admissions with HC, a propensity-matched analysis was performed with a ratio of 1:1 without replacement using a caliper width of 0.01. The absolute standardized difference of <10 % was obtained for most variables after propensity matching. We did not require ethical approval was not required for this study as the NIS database does not contain any identifiable information about patients.

## 3 Results

Propensity-matched obese cohorts admitted for CA, PBS + vs PBS- (n = 1275 each), had patients with comparable demographics with age (mean 58 years, p = 0.112), whites (>70 %, p = 0.024), females (>60 %, p = 0.022), and Medicare enrollees (>40 %, p = 0.386) in both cohorts.

PBS + cohort had lower rates of diabetes (27.8 % vs 36.1 %), hyperlipidemia (33.7 % vs 48.6 %), chronic renal failure (17.3 % vs 22.0 %), chronic pulmonary disease (11.8 % vs 21.2 %), congestive heart failure (12.9 % vs 21.6 %) and higher rates of anemias (18.4 % vs 12.2 %), liver disease (5.1 % vs 2.4 %), depression (12.5 % vs 5.5 %) and alcohol abuse (6.7 % vs 2.4 %) vs. PBS- cohort (p < 0.05). The rates of hypertension (73.7 % vs 75.3 %, p = 0.363), pulmonary circulation disorder (3.5 % vs 3.9 %, p = 0.601), smoking (37.3 % vs 34.9 %, p = 0.216), coagulopathy (13.3% vs 11.8 %, p = 0.232) and drug abuse (2.4 % vs 2 %, p = 0.495) were comparable in both PBS+ and PBS- cohort.

Despite a comparable burden of CVD risk factors, there was no significant difference in all-cause mortality (46.3 % vs 45.1 %, p = 0.551) between the two cohorts. The PBS + cohort was less often transferred routinely (20.4 % vs 22.4 %, p < 0.001) and had a shorter LOS (5 vs 6 days, p < 0.001) with higher hospital charges (27,438 vs 24,298, p = 0.773) compared to the PBS- cohort but statistically insignificant (Table 1).

**Table 1:** Outcomes of Cardiac Arrest Hospitalizations in Obese Patients With vs. Without Prior Bariatric Surgery Status: Propensity-score Matched Analysis.

Variable		No prior bariatric surgery (N = 1275)		Prior Bariatric Surgery (N = 1275)		P
		N	%	N	%	
Age (years) at admission		58 [47–68]		58 [49–65]		0.112
Sex	Male	420	32.9 %	475	37.3 %	0.022
	Female	855	67.1 %	800	62.7 %	
Race	White	920	72.2 %	950	74.5 %	0.024
	Black	290	22.7 %	245	19.2 %	
	Hispanic	45	3.5 %	45	3.5 %	
Non-elective		1145	89.8 %	1120	87.8 %	0.116
Primary expected payer	Medicare	560	43.9 %	560	43.9 %	0.386
	Medicaid	145	11.4 %	125	9.8 %	
	Private including HMO	520	40.8 %	535	42.0 %	
Median household income quartile	0-25th	375	29.4 %	375	29.4 %	0.623
	76-100th	185	14.5 %	185	14.5 %	
Large bed sized hospital admission		700	54.9 %	720	56.5 %	
Location/teaching status of hospital	Rural	50	3.9 %	70	5.5 %	0.019
	Urban non-teaching	330	25.9 %	280	22.0 %	
	Urban teaching	895	70.2 %	925	72.5 %	
Comorbidities						
Hypertension		960	75.3 %	940	73.7 %	0.363
Diabetes Mellitus		460	36.1 %	355	27.8 %	<0.001
Hyperlipidemia		620	48.6 %	430	33.7 %	<0.001
Smoking		445	34.9 %	475	37.3 %	0.216
Alcohol abuse		30	2.4 %	85	6.7 %	<0.001
Drug abuse		25	2.0 %	30	2.4 %	0.495
Renal failure		280	22.0 %	220	17.3 %	0.003
Liver disease		30	2.4 %	65	5.1 %	<0.001
Coagulopathy		150	11.8 %	170	13.3 %	0.232
Congestive heart failure		275	21.6 %	165	12.9 %	<0.001
Valvular heart disease		35	2.7 %	15	1.2 %	0.004
Chronic pulmonary disease		270	21.2 %	150	11.8 %	<0.001
Pulmonary circulation disease		50	3.9 %	45	3.5 %	0.601
Depression		70	5.5 %	160	12.5 %	<0.001
Deficiency Anemias		155	12.2 %	235	18.4 %	<0.001
Outcomes						
All-cause mortality		575	45.1 %	590	46.3 %	0.551
Disposition of patient	Routine	285	22.4 %	260	20.4 %	<0.001
	Transfers to short term hospital	55	4.3 %	60	4.7 %	
	Other transfers, SNF, ICF, etc.	260	20.4 %	200	15.7 %	
	Home health care	100	7.8 %	165	12.9 %	
Length of stay (days), median [IQR]		6 [[3], [4], [5], [6], [7], [8], [9], [10], [11], [12]]		5 [[2], [3], [4], [5], [6], [7], [8], [9], [10]]		<0.001
Cost (USD), median [IQR]		24,298 [15,105–49106]		27,438 [13,632–47431]		0.773

P < 0.05 was considered statistically significant. IQR = interquartile range, HMO= Health Maintenance Organization, SNF = Skilled Nursing Facility, ICF = Intermediate Care Facility.

Obesity was defined as BMI≥30.

Propensity matched analysis was adjusted for these covariates-age at admission, sex, race, payer status, household income quartile, hospital bed size, location/teaching status and region.

## 4 Discussion

The association between obesity and its impact on CVD risk factors seems to be well-defined, and studies comparing the impact of BMI on monozygotic twins in more than 5000 study populations showed an increased incidence of diabetes mellitus without significant impact on the incidence of myocardial infarction and death [9]. Given that genotypically, BMI does not affect mortality outcomes, outcomes of patients with obesity undergoing BAS which can phenotypically benefit patients, shows improved all-cause mortality and decreased incidence of death from CVD causes based on various studies [2,3]. While outcomes for IHCA studied so far focus on resuscitation with quality of cardiac compressions, total time of cardiopulmonary resuscitation [10], there is a lack of data pursuing factors such as patient characteristics associated with outcomes of IHCA. There is conflicting evidence for role of obesity in cardiovascular outcomes. Gupta et al. showed an “obesity paradox” effect in survival with IHCA; however, studies have shown improved cardiovascular outcomes in the PBS cohort as well [11], thus we aimed to analyze if PBS status affected CA developed in hospitalizations on patients with obesity and related mortality outcomes.

In our analysis, we found hospitalizations with CA with PBS + versus PBS- were not affected by demographics such as age, sex, ethnicity, or economic status. In terms of comorbidities, we found that the PBS + cohort had less frequent diabetes mellitus and hyperlipidemia, however, we did not find a significant difference in the prevalence of hypertension. Meta-analysis of long-term effects of BAS showed improved rates of type 2 diabetes mellitus, hypertension, and hyperlipidemia, with improvement being twice as much with gastric bypass as compared to gastric banding [11]. PBS + cohort in our analysis showed similar results for diabetes and hyperlipidemia, however, not in hypertension which could be due to undifferentiated PBS status instead of type of procedure. PBS status is associated with increased rates of anemia such as iron deficiency, vitamin B12 deficiency and other deficits including vitamin D deficiency, micronutrients, and macronutrients like protein malnutrition [12]. Our study coincides with the known findings of higher anemias and alcohol use which is also associated with worse nutritional status.

This is the first national analysis conducted to analyze the direct effect of BAS status in patients with obesity on IHCA outcomes given the limited data available. Studies so far have studied all-cause mortality; however, the effect of PBS status on outcomes of CA has not been studied. Contrary to the well-known benefits of BAS, we found that all-cause mortality in obese patients admitted for CA did not have any improved survival outcomes for hospitalizations with PBS status. Meta-analyses conducted so far have shown mortality benefits from BAS by reducing CVD- and all-cause mortality; however, none of the studies have been randomized to a control group without BAS which is the limitation to proving improved outcomes solely from BAS [13]. This study further strengthens the claim and emphasizes the need for randomized trials to understand the role of BAS and perhaps the role of rigorous CVD burden in improving survival outcomes in patients with obesity.

## 5 Limitations

Limitations of our include inherent constraints of the database like coding or billing errors despite using validated ICD-10 codes. Since this is a retrospective observational study, we could not assess the cause of cardiac arrest especially with post-mortem findings which would confirm causation, if the return of spontaneous circulation was achieved, and the duration between BAS and sentinel CA event to argue causality. We were also unable to assess the severity of obesity, number of years of obesity, amount of improvement in weight after BAS, specific type of BAS offered to the PBS cohort, assess the difference in treatment in both group such as vascular access, airway management, diagnostic procedures which can be typically more prevalent in obese patients, complications associated with the procedure if any and duration between BAS and beneficial effects in terms of cardiovascular disease which were not noted in our study and requires prospective studies for further analysis. We were also unable to provide length of cardiac arrest, comorbid factors leading to cardiac arrest such as presence of coronary artery disease, medication use, etc. Additionally, resuscitation after 10.13039/100004676CA is subject to availability of experts and hemodynamic support available for the patient. Nonetheless, this is the first large-scale study to our knowledge from the US providing preliminary insights into this subject and warrant future longitudinal studies on the subject.

With our analysis, we find that BAS is shown to reduce the overall cardiovascular burden, however, we did not find survival benefit, which could be due to the observational nature of our study. While BAS can offer CVD benefit by decreasing metabolic burden on the patient, post BAS, a prospective long term analysis comparing lifestyle interventions and overall health status may be able to provide strong correlation and potentially prove survival benefit of BAS and/or impact and necessity of continued lifestyle interventions.

## 6 Conclusion

In this propensity-matched analysis, we find PBS status did not improve the survival outcomes in obese patients with CA hospitalizations without taking into account the time interval between BAS and CA hospitalizations. While the study shows a comparable CVD burden in propensity-matched population with PBS when compared to hospitalizations in PBS-, further validating the previous literature, PBS status did not impact survival outcomes in CA hospitalizations in our analysis. Further lifestyle and medical interventions are needed in combination with surgical interventions to control CVD risk factors and perhaps non-CVD comorbidities in patients with obesity with PBS to improve survival outcomes in CA-related hospitalizations.

## CRediT authorship contribution statement

**Rupak Desai:** Conceptualization, Data curation, Formal analysis, Methodology, Project administration, Writing - original draft, Writing - review & editing. **Zainab Gandhi:** Project administration, Visualization, Writing - original draft, Writing - review & editing. **Abhimanyu Ravalani:** Data curation, Writing - original draft, Writing - review & editing. **Kamran Mahfooz:** Data curation, Writing - original draft, Writing - review & editing. **Uvesh Mansuri:** Writing - original draft, Writing - review & editing. **Akhil Jain:** Writing - original draft, Writing - review & editing. **Ankit Vyas:** Supervision, Visualization, Writing - review & editing. **Rajeev Gupta:** Supervision, Visualization, Writing - review & editing. **Carl J. Lavie:** Supervision, Visualization, Writing - review & editing.
